# Estimation of Cardiovascular Risk Using SCORE2, REGICOR and Vascular Age Scales in Spanish Healthcare Workers: A Retrospective Longitudinal Study

**DOI:** 10.3390/healthcare13040375

**Published:** 2025-02-10

**Authors:** Pedro Javier Tárraga Marcos, Pedro Juan Tárraga López, Ángel Arturo López-González, Emilio Martínez-Almoyna Rifá, Hernán Paublini Oliveira, Cristina Martorell Sánchez, José Ignacio Ramírez-Manent

**Affiliations:** 1 Sant Joan University Hospital, 03550 Alicante, Spain; 2 Faculty of Medicine, Castilla la Mancha University, 02071 Albacete, Spain; pjtarraga@sescam.jccm.es; 3 ADEMA-Health Group of University Institute of Health Sciences (IUNICS) of Balearic Islands, 07120 Palma de Mallorca, Spain; emilio@udemax.com (E.M.-A.R.); h.paublini@eua.edu.es (H.P.O.); c.martorell@eua.edu.es (C.M.S.); joseignacio.ramirez@ibsalut.es (J.I.R.-M.); 4 Faculty of Odontology, University School ADEMA-UIB, 07009 Palma de Mallorca, Spain; 5 Health Service of the Balearic Islands, 07003 Palma de Mallorca, Spain; 6 Faculty of Medicine, Balearic Islands University, 07122 Palma de Mallorca, Spain

**Keywords:** cardiovascular risk, mediterranean diet, physical activity, SCORE2, REGICOR, vascular age

## Abstract

**Background/Objectives:** Cardiovascular diseases (CVD) are one of the major public health concerns worldwide due to their high morbidity and mortality rates. This situation has necessitated the development of tools to assess cardiovascular risk (CVR) in order to prevent the onset of CVD. The aim of this study is to assess how sociodemographic variables and health habits are associated with the values of CVR scales, such as REGICOR, SCORE2, and vascular age. **Methodology:** This is a descriptive and cross-sectional study involving 44,939 Spanish healthcare workers, where the association between age, sex, type of work, smoking, physical activity, and Mediterranean diet with CVR scales such as REGICOR, SCORE2, and vascular age was evaluated. **Results:** All the variables analyzed showed an association with the occurrence of moderate-high values in all three CVR scales. Age was the variable that showed the highest odds ratio values: 13.446 (95% CI 11.16–14.77) for REGICOR, 13.90 (95% CI 12.01–15.81) for vascular age, and 14.12 (95% CI 12.01–16.23) for SCORE2. **Conclusions:** The profile of a healthcare worker with the highest risk of presenting moderate-high values on all three CVR scales would be a male over 60 years old, a nursing assistant or orderly, a smoker, sedentary, and with low adherence to the Mediterranean diet.

## 1. Introduction

Cardiovascular diseases (CVD) remain one of the leading causes of morbidity and mortality worldwide [1], necessitating a comprehensive understanding of cardiovascular risk: the probability of experiencing cardiovascular events over a defined time period [2]. This concept has evolved significantly, integrating various risk factors and diagnostic tools to enhance predictive accuracy [3].

Cardiovascular risk refers to the likelihood of developing cardiovascular events, such as myocardial infarction or stroke, within a given timeframe [4]. This risk is influenced by modifiable factors (e.g., smoking, hypertension, hyperlipidemia) [5] and non-modifiable factors (e.g., age, sex, genetic predisposition) [6]. Identifying and quantifying these factors is crucial to implementing preventive strategies and personalizing therapeutic interventions [7].

The systematic evaluation of cardiovascular risk has its roots in the mid-20th century, particularly with the inception of the Framingham Heart Study in 1948. This longitudinal cohort study was pivotal in identifying key CVD risk factors such as hypertension, hypercholesterolemia, and smoking, laying the foundation for multivariable risk assessment models [8].

In the ensuing decades, these models were refined by incorporating additional variables and improving predictive capabilities. For example, the development of the Framingham Risk Score provided clinicians with a practical tool for estimating the 10-year risk of developing coronary artery disease, facilitating a more personalized approach to prevention and treatment [8].

Several diagnostic scales and risk prediction models have been developed to quantify cardiovascular risk, each with unique characteristics and applications, as seen in Table 1.

The concepts of vascular age and cardiac age have emerged as innovative approaches to improve communication and risk prediction. Vascular age represents the biological age of an individual’s vascular system, often reflecting cumulative exposure to risk factors such as smoking, hypertension, and hypercholesterolemia [15]. Similarly, cardiac age estimates the biological age of the heart, integrating traditional risk factors with imaging data, such as coronary artery calcium scores [16].

These metrics are particularly valuable in clinical practice, translating complex risk scores into terms easily understandable by patients. For example, individuals with high cardiovascular risk but no manifest disease may present with a vascular or cardiac age significantly greater than their chronological age. Studies have demonstrated that incorporating these metrics into clinical evaluations enhances patients’ motivation to adopt lifestyle changes and adhere to treatments [17].

Recent research has focused on refining traditional risk factors and exploring novel biomarkers to improve risk prediction. For instance, measures such as ambulatory blood pressure monitoring [18], central hemodynamic dynamics, and concentrations of low-density lipoprotein particles [19] have been investigated for their potential to enhance cardiovascular risk detection. Additionally, the integration of genetic testing has been proposed as a method to provide a more comprehensive assessment of an individual’s cardiovascular risk profile [20].

The aim of this study is to assess the level of cardiovascular risk using various scales such as SCORE2, REGICOR, and vascular age in a large cohort of Spanish healthcare workers.

## 2. Methods

### 2.1. Study Design and Sample Characteristics

This research employed a mixed-methods approach, integrating a retrospective longitudinal study with a cross-sectional descriptive analysis. The study population comprised 44,939 healthcare professionals from diverse regions across Spain. Of these, 14,305 were male (31.8%), while 30,634 were female (68.2%).

Participants were drawn from individuals undergoing mandatory annual occupational health examinations as part of routine employer-provided medical assessments conducted during the study period. The longitudinal component of the study spanned from 2010 to 2019, allowing for an extended analysis of health trends over time.

### 2.2. Inclusion Criteria:

Aged between 18 and 69 years.

Employed by one of the participating companies.

Provided informed consent to participate in the study.

Authorized the use of their data for epidemiological purposes.

**Figure 1:**
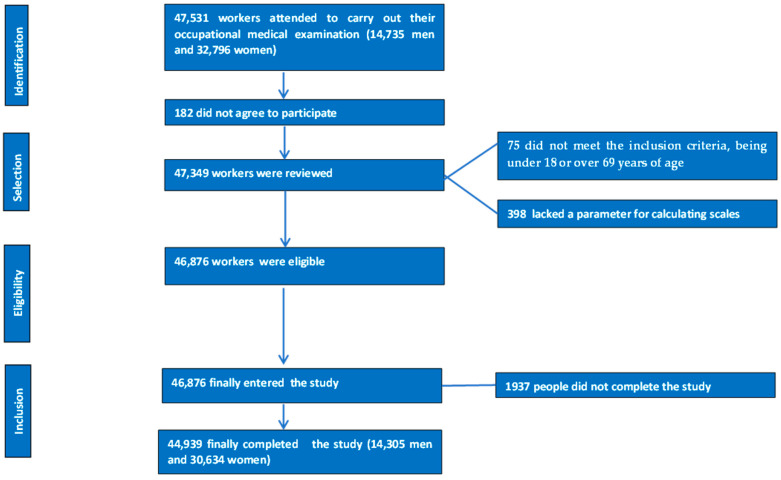
Flowchart detailing the selection and inclusion process for study participants.

The flow chart depicting the selection of study participants is presented in Figure 1.

### 2.3. Data Collection Procedures

Occupational health teams from the collaborating organizations were responsible for data collection, employing standardized methodologies to ensure consistency and reliability across measurements.

Medical History Assessment

Sociodemographic data, including age, sex, and occupational category, were recorded alongside health-related variables such as smoking status, physical activity levels, adherence to a Mediterranean dietary pattern, and perceived stress levels.

Anthropometric and Clinical Measurements

A range of physical and physiological parameters were assessed, including height, weight, and waist and hip circumference, as well as systolic and diastolic blood pressure.

Laboratory Analyses

Biochemical assessments encompassed lipid and liver profiles, along with fasting blood glucose levels, to provide a comprehensive evaluation of metabolic health.

To mitigate potential measurement biases, all procedures were conducted following standardized protocols:

- Height and Weight: Measurements were obtained using a SECA 700 scale and a SECA 220 stadiometer, with participants wearing only light undergarments.
- Circumference Measurements: Waist circumference was assessed using a SECA measuring tape positioned at the midpoint between the lowest rib and the iliac crest, while hip circumference was measured at the widest part of the buttocks, ensuring participants maintained a relaxed standing posture.
- Blood Pressure Monitoring: Blood pressure readings were collected using an OMRON-M3 sphygmomanometer following a 10 min seated rest period. Participants were instructed to abstain from eating, drinking, or smoking for at least one hour prior to measurement. Three readings were taken at one-minute intervals, with the final value calculated as the average of these measurements.

Venous blood samples were obtained after a minimum fasting period of 12 h, refrigerated immediately, and processed at certified reference laboratories within 72 h. The laboratory analyses included:

- Triglyceride, total cholesterol, and glucose quantification via enzymatic methods.
- High-density lipoprotein (HDL) cholesterol determination using precipitation techniques.
- Low-density lipoprotein (LDL) cholesterol estimation via the Friedewald equation, applicable when triglyceride concentrations remained below 400 mg/dL [21].

### 2.4. CVR Scales

The guidelines introduce a new model for estimating cardiovascular risk: SCORE2. This system has been adapted to four regions in Europe based on cardiovascular mortality rates, with Spain categorized among the countries with low vascular risk. The SCORE2 uses non-HDL cholesterol = (total cholesterol − HDL cholesterol) as a metric; therefore, it is extremely important to know the HDL-c values to be able to perform the calculation. The tool allows for calculation of the risk of experiencing major cardiovascular events, such as myocardial infarction, stroke, or death from vascular causes, over the next 10 years for men and women aged 40 to 89 years [22].

To calculate REGICOR, the tables developed by Marrugat were used, which require total cholesterol, HDL cholesterol, systolic and diastolic blood pressure, smoking status, and the presence or absence of diabetes for calculation. In these tables, HDL cholesterol is a factor influencing cardiovascular risk (CVR). Specifically, if HDL cholesterol is <35 mg/dL, the actual risk is approximately the calculated risk multiplied by 1.5. Conversely, if HDL cholesterol is ≥60 mg/dL, the actual risk is approximately the calculated risk multiplied by 0.5. It can be calculated for individuals aged between 35 and 74 years. A low risk was considered if the values were below 5%, moderate risk between 5% and 9.9%, high risk between 10% and 14.9%, and very high risk if the value was greater than or equal to 15% [23].

Vascular age was estimated using the Framingham model, which incorporates multiple clinical and demographic parameters, including sex, chronological age, levels of high-density lipoprotein (HDL) cholesterol and total cholesterol, systolic and diastolic blood pressure, smoking status, presence of diabetes, and the use of antihypertensive medication [24].

The concept of avoidable lost life years (ALLY) is defined as the numerical difference between an individual’s chronological age (CA) and their calculated vascular age (VA) [25]. A moderate ALLY classification was assigned to individuals with a discrepancy of 10 to 17 years, whereas a high ALLY designation was applied to cases where vascular age exceeded chronological age by 18 years or more [26].

### 2.5. Operational Definitions

Professional Categories: Healthcare personnel were classified into four distinct groups: physicians, nurses, health technicians (encompassing laboratory, pathology, and radiology professionals), and nursing assistants or orderlies.

Smoking Status: Individuals were classified as smokers if they reported consuming at least one cigarette per day over the past 30 days or if they had quit smoking within the previous year.

Diabetes mellitus was defined as a fasting blood glucose level exceeding 126 mg/dL or an HbA1c level equal to or greater than 6.5% in two separate tests, or the presence of both altered values in a single analysis. Additionally, individuals with a prior diagnosis of diabetes mellitus or those undergoing pharmacological treatment for the condition were also classified as diabetic.

Adherence to the Mediterranean diet was evaluated using the PREDIMED questionnaire, with high adherence defined as a score of 9 or higher [27].

Physical activity was assessed using the International Physical Activity Questionnaire (IPAQ), which measures frequency, duration, and intensity [27].

### 2.6. Statistical Analysis

A descriptive analysis was conducted for categorical variables, utilizing frequency distributions to summarize the data. The normality of continuous variables was evaluated using the Kolmogorov–Smirnov test, and parametric variables were subsequently summarized using means and standard deviations. Since all continuous variables followed a parametric distribution, a student’s *t*-test was applied to compare mean values between groups, while a chi-square test was employed to examine differences in proportions. To investigate factors associated with metabolic syndrome (MS) and healthy weight (HTW), binary logistic regression models were constructed, with model adequacy assessed via the Hosmer–Lemeshow test. Additionally, a stratified analysis was performed to explore potential confounding variables; however, no significant confounding effects were identified. All statistical analyses were conducted using SPSS software (version 29.0), with a significance threshold set at *p* < 0.05.

### 2.7. Ethical Considerations

The research team has formally committed to adhering to the ethical principles outlined in the Declaration of Helsinki, with a particular emphasis on maintaining participant anonymity and ensuring the confidentiality of collected data. This study received approval from the Ethics and Research Committee of the Balearic Islands (CEI-IB) on 26 November 2020, under protocol number IB 4383/20. Participation in the study was entirely voluntary; individuals were thoroughly informed about the study’s objectives before providing both written and verbal consent. To this end, participants were given an information sheet explaining the study’s purpose, the anonymization process, and the assurance that their identities would not be disclosed in any resulting publications. The research team has pledged not to share any data that could lead to the identification of participants. Furthermore, all individuals involved in the study were granted the right to access, rectify, delete, or contest the use of their personal data. The research team remains fully committed to compliance with Spain’s Organic Law 3/2018, enacted on December 5, which governs personal data protection and the safeguarding of digital rights.

## 3. Results

Table 2 presents the anthropometric, clinical, biochemical, sociodemographic, and health behavior characteristics of the 44,939 healthcare workers included in the study. The mean age of participants was slightly above 41 years. Across all assessed variables, female participants demonstrated lower values compared to their male counterparts. The age distribution of the cohort ranged from 30 to 69 years. Adherence to the Mediterranean diet was reported by 45.8% of male participants and 37.9% of female participants. Regular physical activity was observed among 47.5% of men and 38.9% of women, while smoking prevalence was recorded at 16.1% among men and 15% among women.

Table 3 and Table 4 present the mean values and the prevalence of elevated values for the cardiovascular risk scales (vascular age, SCORE2, and REGICOR) according to different sociodemographic variables (age and type of work) and health habits (smoking, physical activity, and Mediterranean diet) in both sexes. In both cases, whether referring to means or prevalence of high values, the same trend is observed: an increase parallel to age, an increase as socioeconomic status decreases, and an increase among individuals with unhealthy habits (smoking, sedentary lifestyle, and low adherence to the Mediterranean diet). All values (means and prevalence of high values) are higher in men. In all cases, the differences found are statistically significant (*p* < 0.001).

Table 5 presents the results of the multinomial logistic regression. The independent variables include sex, age, type of work, smoking, physical activity, and Mediterranean diet. The reference variables are female sex, age under 30 years for vascular age and REGICOR and under 39 years for SCORE2, being a physician, being a non-smoker, engaging in regular physical activity, and having high adherence to the Mediterranean diet. The dependent variables include moderate-high values for vascular age, SCORE2, and REGICOR.

It is observed that all independent variables increase the risk of moderate-high values on the three cardiovascular risk scales. Among them, the variables with the highest odds ratio (OR) values are advanced age and tobacco use.

Table 6 summarizes the findings of the retrospective longitudinal study conducted from 2010 to 2019. The results indicate that differences in the prevalence of the three CVR scales between the two time periods increase with advancing age. A similar upward trend is observed among individuals with lower socioeconomic status, smokers, those leading sedentary lifestyles, and individuals who do not consistently adhere to a Mediterranean diet.

## 4. Discussion

The cardiovascular risk (CVR) assessed using the REGICOR, SCORE2 scales, and vascular age was associated, in our study, with all the variables analyzed (sex, age, socioeconomic status, tobacco use, physical activity, and adherence to the Mediterranean diet).

Sex emerges as a fundamental determinant of cardiovascular risk when applying these three scales, as distinct patterns of risk were observed between men and women in our study. Consistent with the reviewed literature, men exhibit higher cardiovascular event risks at younger ages compared to women, although this disparity diminishes with advancing age [28]. Models such as the Framingham Risk Score, REGICOR, and SCORE include sex as a key variable, recognizing the differing risk profiles and disease progression between men and women [29]. Men tend to have higher total cholesterol levels, lower HDL cholesterol, and an earlier onset of hypertension, all of which contribute to elevated CVR [30]. In contrast, premenopausal women benefit from the protective effect of estrogen, which supports favorable lipid profiles and vascular function. However, this protective effect wanes after menopause, leading to an increased cardiovascular risk [31].

Studies utilizing REGICOR and SCORE consistently demonstrate that women, despite having initially lower CVR, experience a marked increase in risk after menopause, particularly those with pre-existing risk factors such as hypertension, diabetes, or dyslipidemia [32]. Moreover, women from lower socioeconomic backgrounds and those exposed to adverse social determinants of health may face accelerated vascular health deterioration, which traditional CVR assessments often fail to adequately capture [33].

Age has been identified in our research as one of the most significant predictors of cardiovascular risk, given that the probability of developing cardiovascular disease (CVD) increases with age. Both REGICOR and SCORE2 underscore the importance of age, incorporating it as a central variable in CVR calculations [29]. Models like the Framingham Risk Score similarly recognize that advancing age amplifies the cumulative effects of risk factors such as hypertension, hyperlipidemia, and smoking, thereby increasing the likelihood of cardiovascular events [34].

Age is a critical determinant of future CVR, as the impact of traditional risk factors on atherosclerotic cardiovascular disease (ASCVD) attenuates with aging. The relative weight of CVR decreases due to increasing mortality from non-CV (cardiovascular) causes. In the SCORE2 model, the risk threshold for each category varies by age. Apparently healthy individuals under 50 years of age typically have a low 10-year CVR; however, their lifetime risk may differ significantly from the 10-year estimate. Thus, European guidelines recommend age-specific SCORE2 thresholds for risk classification. For instance, very high risk corresponds to a SCORE ≥ 7.5% in individuals < 50 years, ≥10% in those aged 50–69 years, and ≥15% in those ≥70 years. This approach helps prevent undertreatment in younger individuals and overtreatment in older adults.

While age is a critical determinant of CVR, it also underscores the importance of early intervention. Preventive measures, including lifestyle changes and pharmacological treatments, tend to yield diminishing returns with advancing age [35]. The concept of vascular age, which compares an individual’s CVR to the expected risk for someone of the same chronological age, offers valuable insights into the cumulative impact of risk factors over time. For instance, individuals with multiple risk factors (e.g., hypertension, diabetes, and smoking) may have a vascular age significantly higher than their chronological age, reflecting accelerated cardiovascular aging [36]. REGICOR and SCORE2 account for these dynamics by adjusting CVR predictions based on individual age, highlighting the increasing vulnerability to CVD with advancing years [37].

Socioeconomic status (SES) plays a crucial role in determining cardiovascular risk, as demonstrated in our study, where healthcare workers with lower SES exhibited higher CVR levels. This association may be explained by limited access to healthcare, unhealthy dietary patterns, and greater exposure to environmental stressors, which collectively elevate CVD risk. The relationship between SES and CVR is complex, encompassing both direct and indirect pathways. Individuals with lower SES are more likely to smoke, lead sedentary lifestyles, and consume diets rich in processed foods and low in fruits and vegetables, contributing to higher risks of obesity, hypertension, and dyslipidemia [38].

Although models like REGICOR and SCORE incorporate individual risk factors such as smoking, hypertension, and cholesterol levels, SES is not explicitly included as a variable. However, the influence of SES on cardiovascular health can be inferred through the higher prevalence of these risk factors among individuals with lower SES [39]. Additionally, those in lower SES groups often experience heightened stress levels, which have been shown to increase CVR by contributing to hypertension, metabolic dysfunction, and unhealthy behaviors [40]. Studies employing the REGICOR scale have reported that individuals with lower SES exhibit higher total cholesterol levels and poorer blood pressure control, resulting in higher CVR scores compared to those with higher SES [14].

Smoking is identified in our data as one of the modifiable risk factors most strongly associated with elevated CVR scores across all three scales. This is corroborated by the inclusion of smoking status as a key variable in the Framingham Risk Score, REGICOR, and SCORE2 models. Smoking exacerbates atherosclerosis through mechanisms such as endothelial dysfunction, increased blood pressure, and enhanced platelet aggregation. Smokers also exhibit lower HDL cholesterol and higher total cholesterol levels, further compounding their CVR [41].

The adverse effects of smoking on cardiovascular health are well-documented, with smokers facing significantly higher risks of myocardial infarction, stroke, and other cardiovascular events compared to non-smokers [42]. Notably, the CVR associated with smoking is dose-dependent, increasing with the number of cigarettes smoked per day [43]. Smoking cessation yields substantial cardiovascular benefits, with former smokers experiencing reduced risk over time, although residual risk may persist compared to never-smokers [44].

Physical activity appears to have a protective effect against cardiovascular disease, as evidenced by our findings. This aligns with other studies demonstrating that regular exercise improves cardiovascular fitness, reduces blood pressure, regulates cholesterol levels, and aids in weight management, collectively lowering CVR [45]. Both the Framingham and SCORE models indirectly acknowledge the significance of physical activity by including parameters such as blood pressure and lipid profiles, which are positively influenced by exercise.

Engaging in regular physical activity has been shown to reduce cardiovascular events by enhancing endothelial function, lowering inflammation, and improving the body’s ability to metabolize glucose and lipids [46].

The Mediterranean diet (MD), characterized by high consumption of fruits, vegetables, whole grains, legumes, fish, olive oil, and moderate wine intake, appears to confer significant cardiovascular benefits, according to our research. This finding aligns with studies indicating that adherence to this dietary pattern is associated with lower total cholesterol levels, reduced blood pressure, and a lower incidence of coronary artery disease [47]. The PREDIMED study, a large clinical trial, demonstrated that adherence to the MD significantly reduces the risk of major cardiovascular events [48]. Additionally, a Spanish study found that individuals with high adherence to the MD tend to have lower vascular ages [49].

CVR assessment tools, such as SCORE2 and REGICOR, are widely used to estimate the risk of ASCVD in the general population. However, these tools have several limitations, particularly in their sensitivity to regional and occupational factors. They were developed based on specific population cohorts, which may affect their applicability to other geographic contexts with distinct sociodemographic, genetic, and environmental characteristics. For instance, SCORE2, designed for the European population, may not accurately capture risk in regions with different dietary patterns, prevalence of risk factors, or access to healthcare. Similarly, REGICOR, based on a Spanish cohort, may provide less precise estimates when applied to populations with variations in lifestyle, exposure to environmental pollutants, or disparities in access to CV prevention.

Moreover, these risk models primarily focus on traditional factors such as age, sex, blood pressure, cholesterol levels, and smoking, without adequately incorporating occupational determinants that can significantly influence CVR. Healthcare professionals, shift workers, or individuals exposed to high levels of occupational stress and environmental pollution may develop metabolic alterations, hypertension, and dyslipidemia that are not fully accounted for in these models. The lack of adjustment for occupational factors may lead to an underestimation of actual risk in specific working populations, potentially delaying the implementation of targeted preventive strategies.

Therefore, while SCORE2 and REGICOR remain valuable tools for CVR stratification, it is essential to complement them with individualized clinical assessments and consider adaptations that integrate the influence of environmental and occupational factors in diverse populations.

### Strengths and Limitations

One of the principal strengths of this study is its extensive sample size, encompassing nearly 45,000 healthcare professionals, positioning it as one of the most comprehensive investigations of this population on a global scale. Moreover, it is among the pioneering studies—potentially the first—to concurrently assess cardiovascular risk using three distinct measurement scales across various healthcare worker categories. The broad scope of variables examined, spanning sociodemographic and lifestyle-related factors, in conjunction with the longitudinal nature of the research design, enhances the ability to infer causal relationships.

Nevertheless, certain limitations must be acknowledged. The study does not include unemployed individuals, retirees, those under the age of 18, or adults older than 69, which constrains the generalizability of findings to the broader population. However, the substantial sample size helps mitigate this concern. Another notable limitation is the unavailability of data regarding potential confounding variables, such as existing comorbidities or pharmacological treatments. Additionally, the vascular age estimation formulas employed in this research are derived from a U.S. population, whose dietary patterns may substantially differ from those of the study cohort, potentially influencing the applicability of the results.

## 5. Conclusions

The risk of cardiovascular disease is influenced by a complex interaction of multiple factors, including sex, age, socioeconomic status, smoking, physical activity, and diet. The REGICOR, SCORE, and SCORE2 models, as well as the concept of vascular age, provide valuable tools for assessing an individual’s cardiovascular risk based on these variables. While these models incorporate key risk factors, it is important to recognize that social determinants, such as socioeconomic status, can also play a significant role in shaping cardiovascular risk. Public health interventions aimed at promoting smoking cessation, increasing physical activity, improving diet, and addressing socioeconomic disparities are essential in reducing the burden of cardiovascular disease globally.

## Figures and Tables

**Table 1:** Different cardiovascular risk scales.

Scales	Factors	Estimation
Framingham Risk Score [9]	age, sex, total cholesterol, HDL cholesterol, blood pressure, diabetes, and smoking	10-year risk of MI or death
American Heart Association’s Preventive Risk Equations (PREVENT) [10]	age, sex, total cholesterol, HDL cholesterol, systolic blood pressure, diabetes, smoking, BMI, Glomerular filtration rate, lipid-lowering medication, antihypertensive medication.	10- and 30-year absolute risk of CVD, each of the atherosclerotic CVDs
Pooled Cohort Equations (PCE) [11]	age, sex, race, total cholesterol, HDL cholesterol, systolic blood pressure, diabetes, smoking, antihypertensive medication.	10-year risk for a first atherosclerotic cardiovascular disease
Systematic Coronary Risk Evaluation2 (SCORE2) [12]	age, sex, smoking status, total cholesterol, and systolic blood pressure.	10-year risk of fatal cardiovascular events
QRISK3 [13]	age, sex, race, total cholesterol, HDL cholesterol, systolic blood pressure, diabetes, smoking, antihypertensive medication. chronic kidney disease, heart attack, atrial fibrillation, migraines, Rheumatoid arthritis, systemic lupus, severe mental illness, steroids, BMI	10-year risk of heart attack or stroke
REGICOR [14]	age, sex, blood pressure, cholesterol, and smoking habits	10-year risk of fatal cardiovascular events
Vascular Age and Cardiac Age	smoking, hypertension, and hypercholesterolemia	Aging of the heart or arteries

**Table 2:** Characteristics of the population.

	Men n = 14,305	Women n = 30,634	
	Mean (SD)	Mean (SD)	*p*-Value
Age (years)	41.1 (10.6)	40.4 (10.5)	<0.001
Height (cm)	176.0 (7.5)	162.6 (6.0)	<0.001
Weight (kg)	81.2 (14.5)	63.7 (13.3)	<0.001
Waist circumference (cm)	89.7 (12.6)	76.7 (11.8)	<0.001
Hip circumference (cm)	101.7 (8.8)	99.3 (10.7)	<0.001
Systolic blood pressure (mmHg)	128.2 (13.1)	116.1 (13.8)	<0.001
Diastolic blood pressure (mmHg)	79.9 (10.6)	74.8 (10.1)	<0.001
Total cholesterol (mg/dL)	191.8 (37.2)	187.8 (34.6)	<0.001
HDL-c (mg/dL)	48.9 (11.2)	59.3 (12.8)	<0.001
LDL-c (mg/dL)	120.8 (34.1)	112.1 (30.5)	<0.001
Triglycerides (mg/dL)	111.0 (73.2)	81.7 (47.0)	<0.001
Glucose (mg/dL)	93.6 (18.2)	88.9 (12.4)	<0.001
AST (UI/L)	24.1 (17.2)	18.2 (8.0)	<0.001
ALT (UI/L)	29.0 (36.7)	17.3 (13.7)	<0.001
GGT (UI/L)	30.2 (28.8)	18.1 (18.1)	<0.001
	**N (%)**	**N (%)**	***p*-Value**
<30 years	2400 (16.8)	5984 (19.5)	<0.001
30–39 years	4200 (29.4)	8304 (27.1)	
40–49 years	4512 (31.5)	10128 (33.0)	
50–59 years	2449 (17.1)	5150 (16.8)	
60–69 years	744 (5.2)	1120 (3.6)	
Physicians	5064 (35.4)	5024 (16.4)	<0.001
Nurses	4008 (28.0)	12752 (41.6)	
Health Technicians	1728 (12.1)	4128 (13.5)	
Nurse assistants—Jailers	3505 (24.5)	8782 (28.5)	
Smokers	2304 (16.1)	4592 (15.0)	<0.001
Yes physical activity	6793 (47.5)	11942 (38.9)	<0.001
Yes Mediterranean diet	6534 (45.8)	11413 (37.9)	<0.001
Diabetes	360 (2.5)	464 (1.5)	<0.001

HDL, high density lipoprotein; LDL, low density lipoprotein; AST, aspartate aminotransferase; ALT, alanine aminotransferase; GGT, gamma-glutamyl transpeptidase; SD, standard deviation.

**Table 3:** Mean values of cardiovascular risk scales according sociodemographic variables and healthy habits by sex (student’s *t*-test).

		ALLY VA			SCORE2			REGICOR	
Men	n	Mean (SD)	*p*-Value	n	Mean (SD)	*p*-Value	n	Mean (SD)	*p*-Value
30–39 years	4200	1.6 (6.4)	<0.001	0			2712	2.0 (0.9) *	<0.001
40–49 years	4512	3.3 (7.4)		4512	3.0 (1.0)	<0.001	4512	2.4 (1.1)	
50–59 years	2449	5.3 (9.2)		2449	5.4 (1.9)		2449	4.2 (2.9)	
60–69 years	744	6.9 (11.2)		744	8.1 (1.4)		744	4.9 (2.2)	
Physicians	3960	2.6 (8.1)	<0.001	2832	4.0 (1.8)	<0.001	3456	2.9 (1.9)	<0.001
Nurses	3264	2.9 (7.1)		1416	3.4 (1.4)		2568	2.3 (1.0)	
Health Technicians	1488	3.7 (8.5)		912	4.3 (2.2)		1440	3.1 (1.8)	
Nursing assistants or orderlies	3193	5.3 (9.6)		2545	4.9 (2.3)		2953	3.2 (2.6)	
Non-smokers	10,009	1.8 (7.0)	<0.001	6673	4.0 (1.9)	<0.001	8809	2.7 (1.6)	<0.001
Smokers	1896	13.0 (8.7)		1032	6.3 (2.5)		1608	4.0 (3.2)	
No physical activity	6576	4.7 (8.5)	<0.001	4320	4.6 (2.2)	<0.001	5856	3.1 (2.0)	<0.001
Yes physical activity	5329	2.1 (8.0)		3385	3.9 (2.0)		4561	2.6 (2.0)	
Non Mediterranean diet	6765	4.5 (8.3)	<0.001	4439	4.5 (2.2)	<0.001	6057	3.0 (2.0)	<0.001
Yes Mediterranean diet	5140	2.4 (8.1)		3266	4.0 (2.1)		4360	2.7 (2.1)	
**Women**	**n**	**Mean (SD)**	***p*-Value**		**Mean (SD)**	***p*-Value**		**Mean (SD)**	***p*-Value**
30–39 years	8304	−5.8 (6.0)	<0.001	0			4960	0.9 (0.4)	<0.001
40–49 years	10,128	−4.4 (10.1)		10128	1.4 (0.7)	<0.001	10128	1.5 (1.1)	
50–59 years	5150	4.9 (14.4)		5150	3.1 (1.2)		5150	3.2 (1.8)	
60–69 years	1120	6.3 (15.5)		1120	5.3 (1.6)		1120	4.1 (2.4)	
Physicians	3264	−4.7 (9.8)	<0.001	1920	2.0 (1.4)	<0.001	2400	1.7 (1.5)	<0.001
Nurses	9680	−5.2 (8.7)		5568	1.8 (1.1)		8160	1.4 (1.2)	
Health Technicians	3520	−1.4 (11.8)		2464	2.2 (1.6)		3232	1.9 (1.7)	
Nursing assistants or orderlies	8238	1.3 (13.1)		6446	2.8 (1.6)		7566	2.5 (1.8)	
Non-smokers	20,590	−4.2 (10.3)	<0.001	13566	1.9 (1.2)	<0.001	17710	1.8 (1.5)	<0.001
Smokers	4112	6.7 (11.8)		2832	3.9 (1.7)		3648	2.6 (2.0)	
No physical activity	15,352	−1.5 (12.0)	<0.001	10168	2.2 (1.5)	<0.001	13352	2.0 (1.7)	<0.001
Yes physical activity	9350	−3.9 (10.1)		6230	1.1 (1.0)		8006	1.7 (1.5)	
Non Mediterranean diet	15,841	−1.1 (11.8)	<0.001	10499	2.1 (1.5)	<0.001	13749	2.0 (1.7)	<0.001
Yes Mediterranean diet	8861	−4.4 (10.2)		5899	1.3 (1.1)		7609	1.8 (1.5)	

ALLY, avoidable lost life years; VA, vascular age. SCORE2, Systematic COronary Risk Evaluation 2; REGICOR, Registro Gironí del Corazón; SD, standard deviation.

**Table 4:** Prevalence of high values of cardiovascular risk scales according to sociodemographic variables and healthy habits by sex (chi-square test).

		ALLY VA Mod-High			SCORE2 Mod-High			REGICOR Mod-High	
Men	n	%	*p*-Value	n	%	*p*-Value	n	%	*p*-Value
30–39 years	4200	11.4	<0.001	0			2712	1.8	<0.001
40–49 years	4512	20.2		4512	1.9	<0.001	4512	4.3	
50–59 years	2449	22.9		2449	5.2		2449	31.4	
60–69 years	744	32.4		744	22.3		744	58.1	
Physicians	3960	13.4	<0.001	2832	3.1	<0.001	3456	14.7	<0.001
Nurses	3264	13.0		1416	2.8		2568	2.8	
Health Technicians	1488	16.7		912	10.2		1440	18.8	
Nursing assistants or orderlies	3193	25.6		2545	12.9		2953	20.1	
Non-smokers	10,009	10.3	<0.001	6673	3.1	<0.001	8809	12.5	<0.001
Smokers	1896	50.1		1032	12.6		1608	20.9	
No physical activity	6576	20.7	<0.001	4320	3.0	<0.001	5856	58.6	<0.001
Yes physical activity	5329	12.3		3385	14.5		4561	11.1	
Non Mediterranean diet	6765	19.8	<0.001	4439	2.8	<0.001	6057	55.8	<0.001
Yes Mediterranean diet	5140	13.5		3266	15.1		4360	14.6	
**Women**	**n**	**%**	***p*-Value**	**n**	**%**	***p*-Value**	**n**	**%**	***p*-Value**
30–39 years	8304	2.1	<0.001	0			4960	0	<0.001
40–49 years	10128	8.8		10,128	1.0	<0.001	10,128	1.6	
50-59 years	5150	32.6		5150	3.0		5150	22.6	
60–69 years	1120	38.6		1120	14.2		1120	35.7	
Physicians	3264	5.7	<0.001	1920	2.8	<0.001	2400	7.3	<0.001
Nurses	9680	4.4		5568	2.4		8160	3.1	
Health Technicians	3520	12.4		2464	5.8		3232	7.9	
Nursing assistants or orderlies	8238	20.9		6446	7.4		7566	13.7	
Non-smokers	20,590	7.2	<0.001	13,566	1.9	<0.001	17,710	7.0	<0.001
Smokers	4112	28.6		2832	5.7		3648	13.2	
No physical activity	15,352	12.4	<0.001	10,168	2.2	<0.001	13,352	9.1	<0.001
Yes physical activity	9350	7.2		6230	7.3		8006	6.4	
Non Mediterranean diet	15,841	11.5	<0.001	10,499	2.1	<0.001	13,749	8.8	<0.001
Yes Mediterranean diet	8861	7.9		5899	7.7		7609	6.9	

ALLY, avoidable lost life years; VA, vascular age; SCORE2, Systematic COronary Risk Evaluation 2; REGICOR, Registro Gironí del Corazón.

**Table 5:** Multinomial logistic regression.

	VA Mod-High	SCORE2 Mod-High	REGICOR Mod-High
	OR (95% CI)	OR (95% CI)	OR (95% CI)
Men	2.36 (2.20–2.53)	2.95 (2.50–3.41)	2.17 (1.97–2.38)
30–39 years	1.21 (1.17–1.25)	1	2.71 (2.42–3.00)
40–49 years	4.50 (3.98–5.03)	3.16 (2.70–3.63)	6.93 (6.01–7.86)
50–59 years	8.90 (7.92–9.88)	8.86 (8.00–9.73)	9.10 (8.01–10.20)
60–69 years	13.90 (12.01–15.81)	14.12 (12.01–16.23)	13.46 (11.16–14.77)
Nurses	1.28 (1.17–1.39)	1.12 (1.08–1.16)	1.12 (1.08–1.17)
Health Technicians	1.72 (1.57–1.88)	1.41 (1.28–1.54)	1.43 (1.27–1.59)
Nursing assistants or orderlies	1.94 (1.77–2.12)	1.89 (1.70–2.09)	1.98 (1.73–2.23)
Smokers	7.77 (7.21–8.33)	4.49 (3.89–5.10)	2.12 (1.91–2.33)
No physical activity	1.60 (1.49–1.71)	1.79 (1.60–1.98)	1.81 (1.60–2.03)
Non Mediterranean diet	1.40 (1.33–1.48)	1.56 (1.41–1.71)	1.49 (1.32–1.66)

ALLY, avoidable lost life years; VA, vascular age; SCORE2, Systematic COronary Risk Evaluation 2; REGICOR, Registro Gironí del Corazón; OR, odds ratio.

**Table 6:** Differences in the prevalences of moderate and high values of cardiovascular risk scales between the pre and post periods by sex.

		ALLY VA Mod-High			SCORE2 Mod-High			REGICOR Mod-High		
Men	n	%Pre-Post	Difference %	*p*-Value	%Pre-Post	Difference %	*p*-Value	%Pre-Post	Difference %	*p*-Value
30–39 years	4200	10.6–11.4	6.9					1–6–1.8	9.3	
40–49 years	4512	17.8–20.2	11.8		1.7–1.9	10.2		3.7–4.3	12.6	
50–59 years	2449	19.2–22.9	15.9		4.5–5.2	13.4		25.8–31.4	17.9	
60–69 years	744	25.5–32.4	21.3		18.1–22.3	18.9		44.5–58.1	23.4	
Physicians	5064	12.5–13.4	6.6	<0.001	2.9–3.1	7.6	<0.001	13.7–14.7	6.9	<0.001
Nurses	4008	11.8–13.0	8.9		2.5–2.8	8.9		2.5–2.8	9.2	
Health Technicians	1728	14.3–16.7	14.6		8.7–10.2	14.5		16.0–18.8	14.6	
Nursing assistants or orderlies	3505	21.1–25.6	17.6		10.5–12.9	18.5		16.1–20.1	19.8	
Non-smokers	12,001	9.7–10.3	5.7	<0.001	2.9–3.1	4.6	<0.001	11.8–12.5	5.6	<0.001
Smokers	2304	40.1–50.1	19.9		10.0–12.6	20.9		16.0–20.9	23.6	
No physical activity	7512	16.8–20.7	18.8	<0.001	2.5–3.0	17.9	<0.001	47.2–58.6	19.5	<0.001
Yes physical activity	6793	11.4–12.3	7.2		13.7–14.5	5.4		10.4–11.1	6.4	
Non Mediterranean diet	7771	16.5–19.8	16.8	<0.001	2.3–2.8	16.2	<0.001	45.7–55.8	18.1	<0.001
Yes Mediterranean diet	6534	12.2–13.5	9.2		14.115.1	6.5		13.5–14.6	7.7	
**Women**	**n**	**%**		***p*-Value**	**%**		***p*-Value**	**%**		***p*-Value**
30–39 years	8304	2.0–2.1	5.1					0–0	3.3	
40–49 years	10,128	8.0–8.8	8.6		0.9–1.0	9.5		1.5–1.6	4.8	
50–59 years	5150	29.2–32.6	10.4		2.7–3.0	10.5		20.8–22.6	7.9	
60–69 years	1120	33.3–38.6	13.6		11.9–14.2	16.3		31.1–35.7	12.8	
Physicians	5024	5.4–5.7	5.2	<0.001	2.7–2.8	2.5	<0.001	7.0–7.3	3.5	<0.001
Nurses	12,752	4.1–4.4	6.1		2.3–2.4	2.9		2.9–3.1	4.9	
Health Technicians	4128	11.1–12.4	10.0		5.3–5.8	8.9		7.0–7.9	8.3	
Nursing assistants or orderlies	8782	17.8–20.9	14.6		6.5–7.4	11.8		12.1–13.7	11.7	
Non-smokers	26,094	7.0–7.2	3.2	<0.001	1.8–1.9	2.9	<0.001	6.6–7.0	4.6	<0.001
Smokers	4592	25.3–28.6	11.6		13.1–5.7	16.8		11.4–13.2	13.5	
No physical activity	18,744	11.1–12.4	10.6	<0.001	2.0–2.2	13.5	<0.001	8.0–9.1	11.8	<0.001
Yes physical activity	11,942	6.9–7.2	4.4		6.9–7.3	4.8		6.0–6.4	5.5	
Non Mediterranean diet	19,213	10.4–11.5	10.0	<0.001	1.8–2.1	12.6	<0.001	7.8–8.8	10.9	<0.001
Yes Mediterranean diet	11,413	7.5–7.9	4.9		7.3–7.7	5.3		6.4–6.9	6.7	

ALLY, avoidable lost life years; VA, vascular age; SCORE2, Systematic COronary Risk Evaluation 2; REGICOR, Registro Gironí del Corazón. Pre year 2010. Post year 2019.

## Data Availability

Data are not available due to ethical or privacy restrictions.

## References

[1] Woodruff R.C., Tong X., Khan S.S., Shah N.S., Jackson S.L., Loustalot F., Vaughan A.S. (2024). Trends in Cardiovascular Disease Mortality Rates and Excess Deaths, 2010–2022. Am. J. Prev. Med..

[2] López-Bueno R., Núñez-Cortés R., Calatayud J., Salazar-Méndez J., Petermann-Rocha F., López-Gil J.F., Cruz B.d.P. (2024). Global prevalence of cardiovascular risk factors based on the Life’s Essential 8 score: An overview of systematic reviews and meta-analysis. Cardiovasc. Res..

[3] Olié V., Grave C., Helft G., Nguyen-Thanh V., Andler R., Quatremere G., Pasquereau A., Lahaie E., Lailler G., Verdot C. (2024). Epidemiology of cardiovascular risk factors: Behavioural risk factors. Arch. Cardiovasc. Dis..

[4] Arita V.A., Beigrezaei S., Franco O.H. (2024). Risk factors for cardiovascular disease: The known unknown. Eur. J. Prev. Cardiol..

[5] Ou D., Hu C., Huang S. (2023). Modifiable Risk Factors and Cardiovascular Outcomes. N. Engl. J. Med..

[6] Bowles N.P., He Y., Huang Y.-H., Stecker E.C., Seixas A., Thosar S.S. (2024). Cardiovascular disease risk: It is complicated, but race and ethnicity are key, a Bayesian network analysis. Front. Public Health.

[7] Raman P., Sagadevan Y., Dhanapalan S., Fernandez B.J., Tan S.Y., Appalasamy J.R., Ramadas A. (2024). Lifestyle-Related Risk Factors and Primary Prevention Strategies for Cardiovascular Diseases in a Middle-Income Country: A Scoping Review and Implication for Future Research. J. Prev..

[8] Mahmood S.S., Levy D., Vasan R.S., Wang T.J. (2014). The Framingham Heart Study and the epidemiology of cardiovascular disease: A historical perspective. Lancet.

[9] Sasikumar M., Oommen A.M., Mohan V.R., Gupta P., Rebekah G., Abraham V.J., George K. (2023). Recalibration of the Framingham risk score for predicting 10-year risk of cardiovascular events: A non-concurrent rural cohort study from Tamil Nadu. Indian Heart J..

[10] Khan S.S., Matsushita K., Sang Y., Ballew S.H., Grams M.E., Surapaneni A., Blaha M.J., Carson A.P., Chang A.R., Ciemins E. (2024). Development and Validation of the American Heart Association’s PREVENT Equations. Circulation.

[11] Lloyd-Jones D.M. (2023). The Pooled Cohort Equations and the Test of Time. J. Am. Coll. Cardiol..

[12] Jabczyk M., Nowak J., Mielcarska S., Hudzik B., Wołkowska-Pokrywa K., Świętochowska E., Zubelewicz-Szkodzińska B. (2024). Evaluation of Cardiometabolic Risk in Patients with Non-Functioning Adrenal Adenomas Using the Systematic Coronary Risk Evaluation 2 (SCORE2) and the Systematic Coronary Risk Evaluation 2-Older Persons (SCORE2-OP) Algorithms. Med. Sci. Monit..

[13] Hippisley-Cox J., Coupland C., Brindle P. (2017). Development and validation of QRISK3 risk prediction algorithms to estimate future risk of cardiovascular disease: Prospective cohort study. BMJ.

[14] Ramírez-Manent J.I., Tomás-Gil P., Coll Villalonga J.L.L., Marti-Lliteras P., López-González A.A., Paublini H. (2024). Association between atherogenic dyslipidemia and lipid triad with cardiovascular risk scales in 418.343 Spanish workers. Acad. J. Health Sci..

[15] Groenewegen K., Ruijter H.D., Pasterkamp G., Polak J., Bots M., Peters S.A. (2016). Vascular age to determine cardiovascular disease risk: A systematic review of its concepts, definitions, and clinical applications. Eur. J. Prev. Cardiol..

[16] Climie R.E., Bruno R.M., Hametner B., Mayer C.C., Terentes-Printzios D. (2020). Vascular Age Is Not Only Atherosclerosis, it Is Also Arteriosclerosis. J. Am. Coll. Cardiol..

[17] Martínez-Díaz A.M., Palazón-Bru A., la Rosa D.M.F.-D., Ramírez-Prado D., Llópez-Espinós P., Beneyto-Ripoll C., Gil-Guillén V.F. (2019). A cardiovascular risk score for hypertensive patients previously admitted to hospital. Eur. J. Cardiovasc. Nurs..

[18] Huang Q.-F., Yang W.-Y., Asayama K., Zhang Z.-Y., Thijs L., Li Y., O’brien E., Staessen J.A. (2021). Ambulatory Blood Pressure Monitoring to Diagnose and Manage Hypertension. Hypertension.

[19] Hoogeveen R.C., Ballantyne C.M. (2021). Residual Cardiovascular Risk at Low LDL: Remnants, Lipoprotein(a), and Inflammation. Clin. Chem..

[20] Tasdighi E., Adhikari R., Almaadawy O., Leucker T.M., Blaha M.J. (2024). LP(a): Structure, Genetics, Associated Cardiovascular Risk, and Emerging Therapeutics. Annu. Rev. Pharmacol. Toxicol..

[21] Pottegård A., Gerdes L.U., Wetche J.L., Thompson W. (2024). Does LDL-C determination method affect statin prescribing for primary prevention? A register-based study in Southern Denmark. Eur. Heart J. Cardiovasc. Pharmacother..

[22] Manzanero R.Z., López-González A.A., Tomás-Gil P., Paublini H., Martínez-Jover A., Ramírez-Manent J.I. (2024). Determination of car-diometabolic risk scales in 7.962 hotel receptionists. Acad. J. Health Sci..

[23] Manzanero R.Z., López-González A.A., Tomás-Gil P., Paublini H., Martínez-Jover A., Ramírez-Manent J.I. (2023). Estimation of cardi-ometabolic risk in 25.030 Spanish kitchen workers. Acad. J. Health Sci..

[24] Kucharska-Newton A.M., Stoner L., Meyer M.L. (2019). Determinants of Vascular Age: An Epidemiological Perspective. Clin. Chem..

[25] Cuende J.I. (2018). Vascular Age, RR, ALLY, RALLY and Vascular Speed, Based on SCORE: Relations Between New Concepts of Car-diovascular Prevention. Rev. Esp. Cardiol..

[26] Montero Muñoz N., López-González A.A., Tomás-Gil P., Martínez Jover A., Paublini H., Ramírez Manent J.I. (2023). Relationship between sociodemographic variables and tobacco consumption with vascular age values using the Framinghan model in 336,450 spanish workers. Acad. J. Health Sci..

[27] Mestre-Font M., Busquets-Cortés C., Ramírez-Manent J.I., Tomás-Gil P., Paublini H., López-González A.A. (2024). Influence of socio-demographic variables and healthy habits on the values of overweight and obesity scales in 386,924 Spanish workers. Acad. J. Health Sci..

[28] Rajendran A., Minhas A.S., Kazzi B., Varma B., Choi E., Thakkar A., Michos E.D. (2023). Sex-specific differences in cardiovascular risk factors and implications for cardiovascular disease prevention in women. Atherosclerosis.

[29] Mancini G.B.J., Ryomoto A., Yeoh E., Brunham L.R., Hegele R.A. (2024). Recommendations for statin management in primary prevention: Disparities among international risk scores. Eur. Heart J..

[30] The Global Cardiovascular Risk Consortium (2023). Global Effect of Modifiable Risk Factors on Cardiovascular Disease and Mortality. New Engl. J. Med..

[31] El Khoudary S.R., Aggarwal B., Beckie T.M., Hodis H.N., Johnson A.E., Langer R.D., Limacher M.C., Manson J.E., Stefanick M.L., Allison M.A. (2020). Menopause Transition and Cardiovascular Disease Risk: Implications for Timing of Early Prevention: A Scientific Statement From the American Heart Association. Circulation.

[32] Álvarez-Fernández C., Romero-Saldaña M., Álvarez-López C., Molina-Luque R., Molina-Recio G., Vaquero-Abellán M. (2021). Gender differences and health inequality: Evolution of cardiovascular risk in workers. Arch. Environ. Occup. Health.

[33] Ghaed S.G., Gallo L.C. (2007). Subjective social status, objective socioeconomic status, and cardiovascular risk in women. Health Psychol..

[34] Shi H., Ge M.-L., Dong B., Xue Q.-L. (2021). The Framingham risk score is associated with incident frailty, or is it?. BMC Geriatr..

[35] Ciumărnean L., Milaciu M.V., Negrean V., Orășan O.H., Vesa S.C., Sălăgean O., Iluţ S., Vlaicu S.I. (2021). Cardiovascular Risk Factors and Physical Activity for the Prevention of Cardiovascular Diseases in the Elderly. Int. J. Environ. Res. Public Health.

[36] Alves-Cabratosa L., Elosua-Bayés M., Martí-Lluch R., Blanch J., Tornabell-Noguera È., Garcia-Gil M., Ponjoan A., Grau M., Ribas-Aulinas F., Zacarías-Pons L. (2024). Cardiovascular Risk Age Reflects Arterial Status: Middle-Aged People Showed Equivalent Arterial Stiffness to Older People in the Same Risk Category. J. Atheroscler. Thromb..

[37] SCORE2-OP Working Group and ESC Cardiovascular Risk Collaboration (2021). SCORE2-OP risk prediction algorithms: Estimating incident cardiovascular event risk in older persons in four geographical risk regions. Eur. Heart J..

[38] Paudel S., Ahmadi M., Phongsavan P., Hamer M., Stamatakis E. (2023). Do associations of physical activity and sedentary behaviour with cardiovascular disease and mortality differ across socioeconomic groups? A prospective analysis of device-measured and self-reported UK Biobank data. Br. J. Sports Med..

[39] He J., Zhu Z., Bundy J.D., Dorans K.S., Chen J., Hamm L.L. (2021). Trends in Cardiovascular Risk Factors in US Adults by Race and Ethnicity and Socioeconomic Status, 1999–2018. JAMA.

[40] Cundiff J.M., Bennett A., Carson A.P., Judd S.E., Howard V.J. (2022). Socioeconomic status and psychological stress: Examining intersection with race, sex and US geographic region in the REasons for Geographic and Racial Differences in Stroke study. Stress. Health.

[41] Aguiló Juanola M.C., López-González A.A., Tomás-Gil P., Paublini H., Tárraga-López P.J., Ramírez-Manent J.I. (2024). Influence of tobacco consumption and other variables on the values of different cardiovascular risk factors in 418,343 Spanish workers. Acad. J. Health Sci..

[42] Rosoff D.B., Smith G.D., Mehta N., Clarke T.-K., Lohoff F.W. (2020). Evaluating the relationship between alcohol consumption, tobacco use, and cardiovascular disease: A multivariable Mendelian randomization study. PLoS Med..

[43] Kondo T., Nakano Y., Adachi S., Murohara T. (2019). Effects of Tobacco Smoking on Cardiovascular Disease. Circ. J..

[44] Delgado G.E., Krämer B.K., März W., Hellstern P., Kleber M.E., Leipe J. (2021). Immune Status and Mortality in Smokers, Ex-smokers, and Never-Smokers: The Ludwigshafen Risk and Cardiovascular Health Study. Nicotine Tob. Res..

[45] Lavie C.J., Ozemek C., Carbone S., Katzmarzyk P.T., Blair S.N. (2019). Sedentary Behavior, Exercise, and Cardiovascular Health. Circ. Res..

[46] Torres García J.C., Tárraga López P.J., Ramírez Gallegos I., Tárraga Marcos A., López González A.A., Ramírez-Manent J.I. (2024). Relationship between physical activity and cardiovascular risk: A systematic review. Acad. J. Health Sci..

[47] Laffond A., Rivera-Picón C., Rodríguez-Muñoz P.M., Juárez-Vela R., de Viñaspre-Hernández R.R., Navas-Echazarreta N., Sánchez-González J.L. (2023). Mediterranean Diet for Primary and Secondary Prevention of Cardiovascular Disease and Mortality: An Updated Systematic Review. Nutrients.

[48] Díez-Espino J., Buil-Cosiales P., Babio N., Toledo E., Corella D., Ros E., Fitó M., Gómez-Gracia E., Estruch R., Fiol M. (2020). Impact of Life’s Simple 7 on the incidence of major cardiovascular events in high-risk Spanish adults in the PREDIMED study cohort. Rev. Espanola Cardiol..

[49] Gómez-Sánchez L., Gómez-Sánchez M., García-Ortiz L., Agudo-Conde C., Lugones-Sánchez C., Gonzalez-Sánchez S., Rodríguez-Sánchez E., Gómez-Marcos M.A. (2024). The Relationship between the Mediterranean Diet and Vascular Stiffness, Metabolic Syndrome, and Its Components in People over 65 Years of Age. Nutrients.

